# Investigation of Phenolic Resin-Modified Asphalt and Its Mixtures

**DOI:** 10.3390/ma17020436

**Published:** 2024-01-16

**Authors:** Lieguang Wang, Lei Wang, Junxian Huang, Mingfei Wu, Kezhen Yan, Zirui Zhang

**Affiliations:** 1 Zhejiang East China Engineering Consulting Co., Ltd., Hangzhou 310030, China; 2 School of Civil Engineering, Hunan University, Changsha 410082, China

**Keywords:** PF-modified asphalt, viscoelastic properties, rheological characteristics, mixture, high-temperature stability

## Abstract

This study comprehensively examines the influence of phenol-formaldehyde resin (PF) on the performance of base asphalt and its mixtures for road applications, emphasizing its innovative use in enhancing pavement quality. Optimal PF content was determined through the evaluation of standard indicators and rotational viscosity. In-depth analyses of PF-modified asphalt’s high- and low-temperature rheological properties and viscoelastic behavior were conducted using dynamic shear rheometers and bending beam rheometers. Aging resistance was assessed through short-term aging and performance grade (PG) grading. Moreover, Marshall and water stability tests were performed on PF-modified asphalt mixtures. Findings indicate that the uniform dispersion of PF particles effectively inhibits asphalt flow at high temperatures, impedes oxygen penetration, and delays the transition from elasticity to viscosity. These unique properties enhance the high-temperature stability, rutting resistance, and aging resistance of PF-modified asphalt. However, under extremely low temperatures, PF’s brittleness may impact asphalt flexibility. Nonetheless, the structural advantages of PF-modified asphalt, such as improved mixture density and stability, contribute to enhanced high-temperature performance, water stability, adhesion, and freeze–thaw cycle stability. This research demonstrates the feasibility and effectiveness of using PF to enhance the overall performance of base asphalt and asphalt mixtures for road construction.

## 1. Introduction

Petroleum asphalt, known for its high viscosity, is widely employed as a binder in road constructions due to its economic advantages, noise reduction capabilities, facilitation of a comfortable driving experience, and ease of use in construction compared with cement road surfaces [1,2]. However, as the global economy rapidly evolves, conventional asphalt pavements are continually plagued by issues such as cracking, ruts, potholes, and surface deformation, rendering them inadequate for meeting the demands of today’s road traffic loads and the harsh and ever-changing natural environment [3,4,5]. Consequently, there is an urgent need to further enhance the performance of asphalt in highway construction.

The use of polymers to enhance the performance of asphalt binders and thereby improve asphalt pavement quality is a popular direction for current research [6,7,8]. For instance, the styrene-butadiene-styrene (SBS) block copolymer, with its strength and elasticity that are derived from a three-dimensional network of crosslinked molecules, has shown significant potential. The polystyrene end blocks in the SBS copolymer impart strength to the polymer, while the polybutadiene rubber matrix provides exceptional viscosity [9]. Researchers like Danial Mirzaiyan et al. [10] have explored the impact of SBS on the fatigue response of asphalt mixtures through indirect tensile fatigue tests (ITFTs), discovering that SBS significantly enhances fatigue resistance by swelling crosslinking in the saturated and aromatic fractions of asphalt. Faheem Sadiq Bhat et al. [11] have confirmed through linear amplitude sweep tests that SBS additions notably improve the high-temperature stability of asphalt binders, reducing the risk of pavement cracking. Simultaneously, a growing number of researchers are exploring rubber modifiers, such as crumb rubber, to enhance pavement performance [12,13]. Peerapong Jitsangiam et al. [14] have found that rubber from recycled tires effectively improves the rutting resistance of base asphalt at high temperatures. Unlike elastomers, Aboelkasim Diab et al. [15] have shown that using ethylene-vinyl acetate (EVA), which is a thermoplastic polymer, to modify asphalt significantly boosts its flexibility and crack resistance at low temperatures. This improvement is closely related to the content and molecular weight of vinyl acetate in EVA. The increase in vinyl acetate content lowers the glass transition temperature (Tg) of EVA, maintaining its flexibility even at lower temperatures.

Despite these advancements, these polymers face challenges in terms of high costs, environmental concerns, and performance limitations under specific conditions [16,17,18]. For instance, Ali Behnood et al. [19] found that polymer-modified asphalt is 1.5 times costlier than traditional asphalt, leading to a 20% increase in the cost of the modified mixtures. SK Sohel Islam et al. [20] have observed that the degradation of SBS at high temperatures significantly reduces the Marshall stability, indirect tensile strength, fatigue, and rutting resistance of asphalt mixtures regardless of the grade and source of the base asphalt. Xiong et al. [21] noted that achieving a high performance in SBS-modified asphalt not only requires chemical adjustments to restore its properties but also the reconstruction of the polymer network through the chemical structure of products from SBS degradation products. Nonde Lushinga et al. [22] have found that enhancing the compatibility of rubber particles with asphalt requires microwave radiation to break the sulfur network on the rubber particle surface for depolymerization and desulfurization. These polymers’ applications are limited by costs, aging, and compatibility issues [23,24]. Simultaneously, Mukul Rathore et al. [25] pointed out that while common polymer modifiers enhance asphalt’s rut resistance, they also significantly increase its viscosity. To reduce production temperatures and promote energy saving and emission reduction, warm mix asphalt additives are often incorporated. Therefore, from both engineering and economic perspectives, selecting appropriate modifiers that balance desired performance with practical application values becomes crucial.

Phenol-formaldehyde resin (PF), as a binding agent derived from the condensation reaction of phenol (C_6_H_5_OH) and formaldehyde (HCHO), is characterized by readily available raw materials, cost-effective production, and a straightforward manufacturing process [26,27]. This compound’s chemical structure exhibits a remarkable stability that confers resistance to oxidation and decomposition, thereby bestowing PF with robust anti-aging capabilities [28]. The three-dimensional polymeric network formed through condensation reactions is particularly noteworthy; it generates a multitude of stable chemical bonds, thereby contributing to the high hardness and strength of phenol-formaldehyde resin [29,30]. As such, PF exhibits superior mechanical properties along with heat and cold resistance, dimensional stability, water stability, and flame resistance, thus making it a versatile material widely applied across various fields [31]. Li et al. [32] discovered excellent compatibility in PF when incorporated into styrene-butadiene rubber (SBR)-modified asphalt. These polar functional groups can form interactions with the polar molecules present in asphalt, facilitating an effective and uniform dispersion of PF within the asphalt mixtures [33]. The ideal internal structure of PF-modified asphalt should consist of a network structure formed through the cross-linking of high binding strength PF and asphalt molecules, leveraging the high-temperature resistance and load-buffering attributes of PF [34]. Despite these promising features, the academic community is yet to exhaustively explore the influence of PF on the mechanical properties of base asphalt and mixed materials. In particular, the current literature provides limited insight into the effects of varying PF contents on these properties.

Therefore, this study systematically evaluated the impact of different PF levels on the conventional mechanical properties, high- and low-temperature rheological properties, and aging resistance of the base asphalt. We also investigated the microstructure and mode of action of PF within the asphalt binder. It uncovered the governing rules of PF on the mechanical properties of base asphalt and, both innovatively and for the first time, correlated the influence mechanism of PF-modified asphalt binders on the stability and resistance to water damage of their mixtures. This research aims to explore the potential of integrating PF into asphalt to enhance pavement performance and extend pavement life, thereby providing novel solutions for sustainable road construction.

## 2. Materials and Preparation

### 2.1. Asphalt

In this study, we selected the domestically produced Baoli 70# road petroleum asphalt. The various performance parameters were tested in accordance with the “ASTM specifications for asphalt binder properties”, as detailed in Table 1.

### 2.2. PF

The phenol-formaldehyde resin (PF) used in this study was sourced from Hebei Zetian Chemical Co., Ltd. (Hengshui, China). Relevant parameters are presented in Table 2.

### 2.3. Aggregates

In accordance with the aforementioned standard, limestone aggregate sourced from Yunzhong Technology Zijing Factory in Changsha, China, was tested. Table 3, Table 4 and Table 5 present the indices for coarse and fine aggregates obtained from the experiment.

### 2.4. Preparation of PF-Modified Asphalt

The preparation of PF composite-modified asphalt was performed according to the method proposed by the previous scholars from [47]. Firstly, the base asphalt was heated to a molten state at 145 °C. Subsequently, varying proportions of PF (representing 1%, 2%, 3%, 4%, and 5% of the total weight of the asphalt) were gradually introduced into a low-speed mixer at 145 °C. The mixture was then subjected to high-speed shearing at a rate of 4000 rpm for 45 min at 165 °C followed by a final stirring at 2000 rpm for 30 min. After this process, the mixture was allowed to cool, yielding the PF-modified asphalt. The preparation procedure for the 70# base asphalt is consistent with the points provided above, with a PF dosage of 0%.

## 3. Mixture Design and Test Method

### 3.1. Design and Preparation of PF-Modified Asphalt Mixtures

#### 3.1.1. Design of Aggregate Gradation

**Figure 1:**
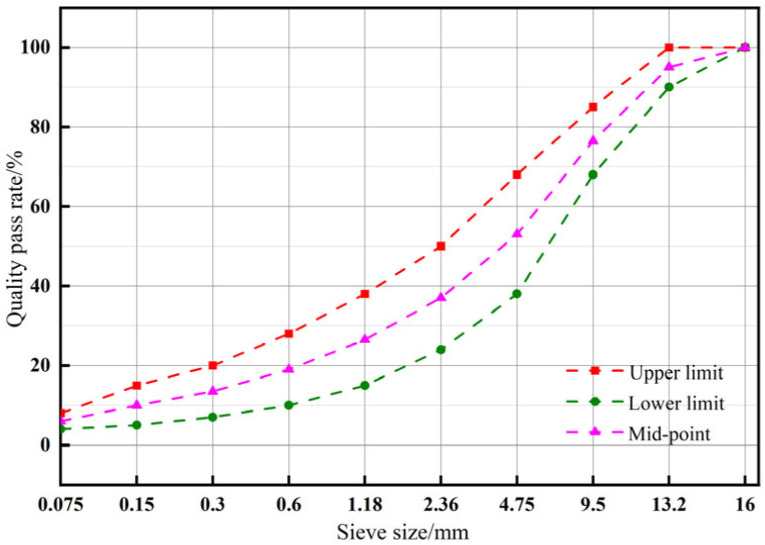
Asphalt mixture grading curve.

The dense-graded AC-13C was selected for the preparation of the asphalt mixture. The gradation curve of the aggregates is shown in Figure 1, and the median curve of the gradation was determined as the design for the aggregate gradation.

#### 3.1.2. Determination of Mixing and Compaction Temperature

**Figure 2:**
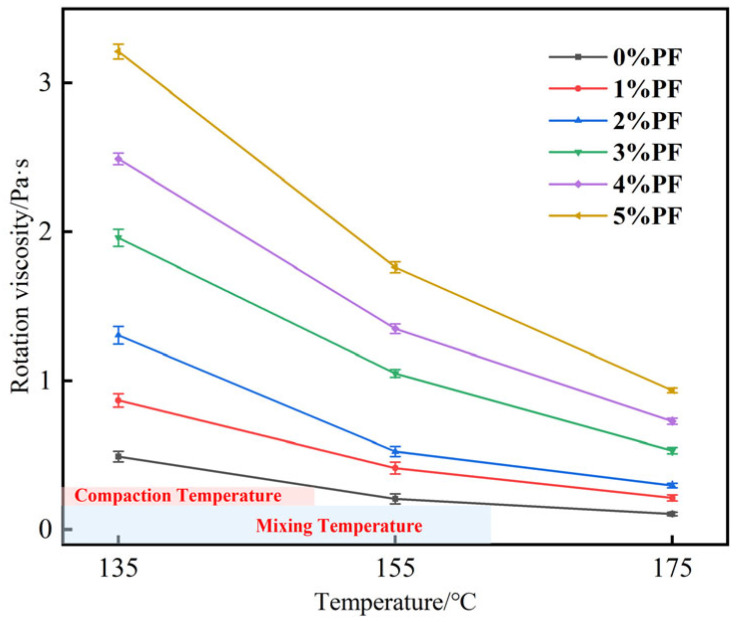
Rotational viscosity diagram for different asphalts.

In accordance with the requirements of the “D6926/D6926M—standard practice for preparation of bituminous specimens using Marshall apparatus”, the mixing and compaction temperatures were determined based on the corresponding temperatures of 0.17 Pa·s ± 0.02 Pa·s and 0.28 Pa·s ± 0.03 Pa·s, respectively (Figure 2). Therefore, the mixing and compaction temperatures for the matrix asphalt were 160 °C and 150 °C, respectively. When the mixing and compaction temperatures of modified asphalt with a PF content range of 2–5% at 175 °C; the highest temperature of 175 °C was selected.

#### 3.1.3. Determination of the Optimal Asphalt–Aggregate Ratio and Volumetric Parameters

According to the ASTM D2726 and AASHTO T166-07 standards [48], Marshall specimens were prepared, selecting five different mass percentages of 4%, 4.5%, 5%, 5.5%, and 6%. The Marshall stability test was used to measure the bulk relative density (*γf*), stability (*MS*), flow value (*FL*), void ratio (*VV*), aggregate void ratio (*VMA*), and effective asphalt saturation (*VFA*). As shown in Table 6, the optimum asphalt content (*OAC*) for different asphalt mixtures was calculated through Formulas (1) and (2).

(1) $${OAC}_{1}=(a_{1}+a_{2}+a_{3}+a_{4})/4$$

(2) $$OAC_{2}=({OAC}_{max}+{OAC}_{min})/2$$

In the formulas, *a*_1_ represents the asphalt content corresponding to the maximum value of *γf*, *a*_2_ corresponds to the asphalt content at which maximum *MS* is achieved, *a*_3_ is the asphalt content at the median value of *VV*, *a*_4_ indicates the asphalt content at the median value of *VFA*, *OAC_min_* is the minimum range of optimum asphalt content as specified by the standard criteria, and *OAC_max_* is the maximum range as specified by the standard criteria, as referred to in Table 6.

### 3.2. Test Method

#### 3.2.1. Mechanical Properties of Asphalt Binders

Firstly, the durability, stability, and resistance to deformation of different asphalt materials are assessed using the three major indicators. The optimal dosage range of PF was determined through the Brookfield viscosity test. The dynamic shear rheometer (DSR) is utilized to measure the phase angle (*δ*) and complex modulus (G*) of asphalt at varying strains and frequencies, thereby evaluating its high-temperature rheological performance. By subjecting the asphalt to loading at different stress levels and frequencies, the multiple stress creep recovery test (MSCR) simulates complex traffic loads and climate variations experienced on roads. The bitumen bending beam rheometer (BBR) method is employed to impose constant shear stresses on various types of asphalt at temperatures of −12 °C and −18 °C, thereby generating the bending beam rheometer stiffness (S) and creep rate (m) and further evaluating the asphalt’s deformation behavior and brittle characteristics under low-temperature conditions. By utilizing the performance grade (PG) grading system for different asphalt types, a more holistic and scientifically informed prediction of the fundamental properties and application scenarios of PF-modified asphalt can be achieved. Lastly, the ratio of the rutting factor (G*/sin*δ*) for different asphalts before and after short-term aging is calculated. The impact of PF on the chemical composition of asphalt binders and its micro-morphology within the asphalt binder were characterized using fourier transform infrared spectroscopy (FT-IR) and fluorescence microscopy (FM).

#### 3.2.2. Mechanical Properties of Asphalt Mixture

The Marshall test is utilized to quantify both the compressive strength and deformation behavior of the mixture, thereby determining the optimal asphalt–aggregate ratio and mixture composition. Moreover, it serves to simulate and assess the stability and endurance of asphalt mixtures under varying traffic loads. The immersion Marshall test evaluates the wet stability and resistance to erosion of the water in the mixture. Concurrently, the freeze–thaw splitting test identifies the mixture’s vulnerability to cracking and its ability to withstand freeze–thaw cycles, thus guaranteeing the robustness and dependability of roads exposed to adverse climatic conditions. The above-mentioned tests all adhere to the ASTM standards for asphalt and asphalt mixture testing. For each type of sample, three parallel experiments were conducted, and the standard error was calculated. Error bars have been annotated in the figure to indicate this variability.

## 4. Results and Discussion

### 4.1. Empirical Indicator Testing

**Figure 3:**
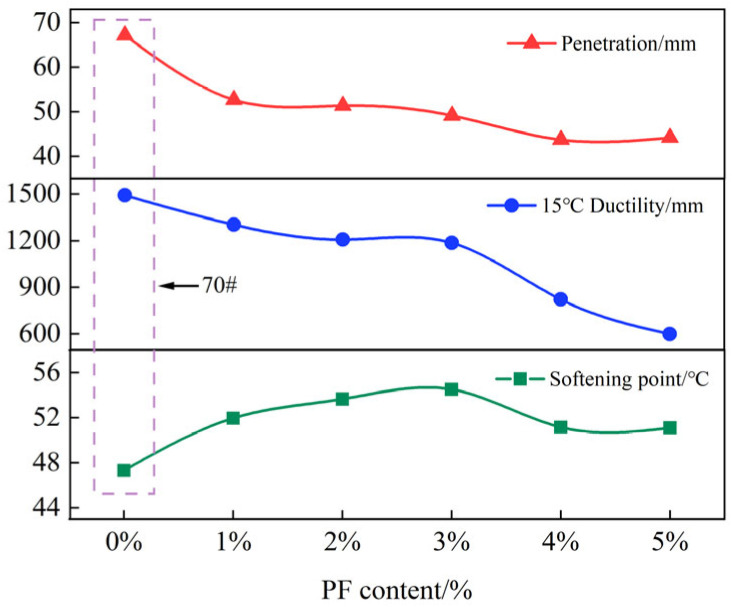
The three major indicators of different types of PF doping.

As can be seen from Figure 3, with the addition of PF, the penetration of asphalt gradually decreases as the amount of PF increases. The ductility of asphalt shows the same trend, and the rate of decrease accelerates. The softening point of PF-modified asphalt initially rises and then falls as the PF content increases, indicating that compatibility issues between PF and asphalt become prominent when the amount of PF is excessive. Overall, the incorporation of PF results in decreased penetration and ductility and an increased softening point of the asphalt. Compared with the base asphalt, PF-modified asphalt has improved elasticity and high-temperature performance but reduced plasticity. These findings are consistent with other research results on PF-modified asphalt [49].

### 4.2. Rotational Viscosity Testing

**Figure 4:**
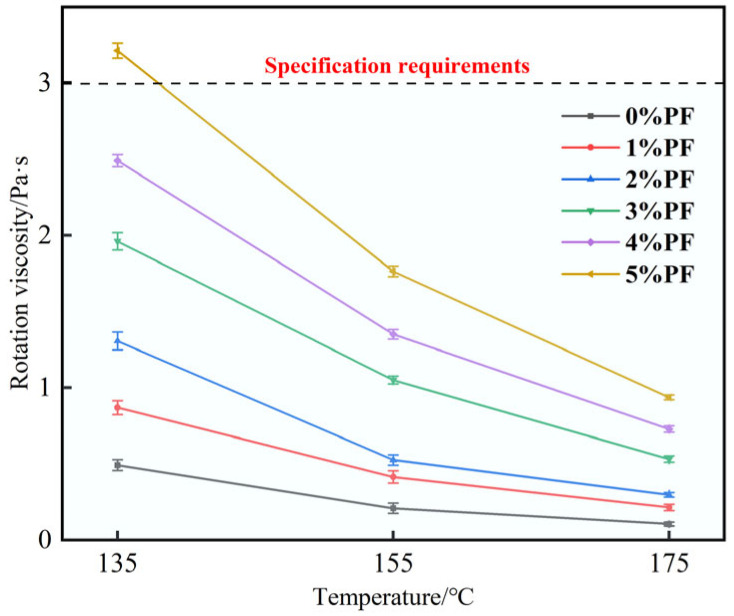
PF-modified asphalt rotational viscosity data graph.

As can be seen from Figure 4, as the temperature rises the intermolecular binding strength of the asphalt decreases and its fluidity increases. Meanwhile, the viscosity values of PF-modified asphalt are all higher than that of the 70# base asphalt, and they increase with the addition of PF. When the PF content is between 2 and 3%, the increase in asphalt’s viscosity is the most significant result. However, the rotational viscosity of 5% PF-modified asphalt at 135 °C exceeds 3.0 Pa·s, suggesting that the use of 5% PF-modified asphalt to prepare asphalt mixtures may lead to uneven mixing and an unstable performance due to excessively high viscosity. The results indicate that the appropriate addition of PF improves the adhesion and high-temperature flow deformation resistance of the asphalt.

By combining the results from the three main indicators and the rotational viscosity test, it can be observed that 2% PF- and 3% PF-modified asphalts have numerous advantages. They exhibit an improved high-temperature performance, a lesser degree of decrease in ductility, and an increase in rotational viscosity while also meeting the specification requirements for mixing viscosity.

### 4.3. Temperature Sweep Testing

**Figure 5:**
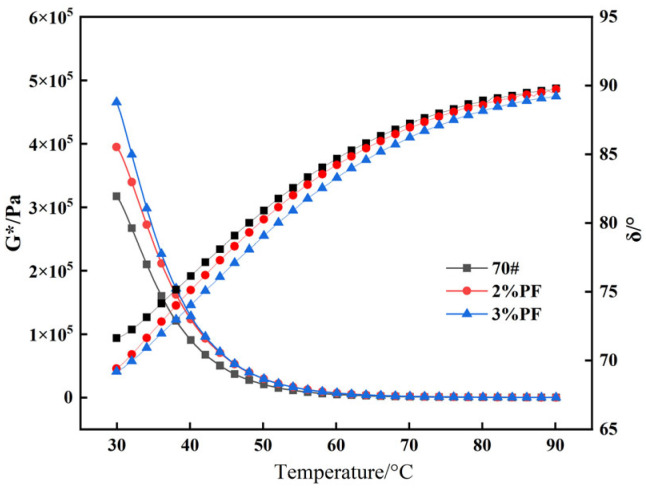
Different asphalt temperature scanning test results.

The rheological properties of the asphalt were evaluated using the DSR test in accordance with the ASTM standard D7175. As can be seen from Figure 5, the G* of different types of asphalt decreases with the increase in temperature, and the rate of decrease slows down at 60 °C. This suggests that the deformation resistance of asphalt decreases under the continuous action of temperature and repeated loads, and all types of asphalt essentially lose their deformation resistance upon reaching 90 °C. The G* curve of PF-modified asphalt is higher than that of the base asphalt, indicating that PF has a positive effect on the high-temperature shear deformation resistance of asphalt.

Further evaluation of the viscoelastic boundary state of asphalt specimens during the experiment through the *δ* revealed that the *δ* value increases with a rising temperature, indicating that the asphalt in question gradually tends toward transitioning to a viscous state. The addition of PF alleviates the degree to which asphalt transitions to a viscous state in a gradually heating environment. The *δ* value of the base asphalt approaches 90° at 90 °C, indicating that the elastic properties of the base asphalt are essentially lost and irreversible deformation is gradually accumulated.

**Figure 6:**
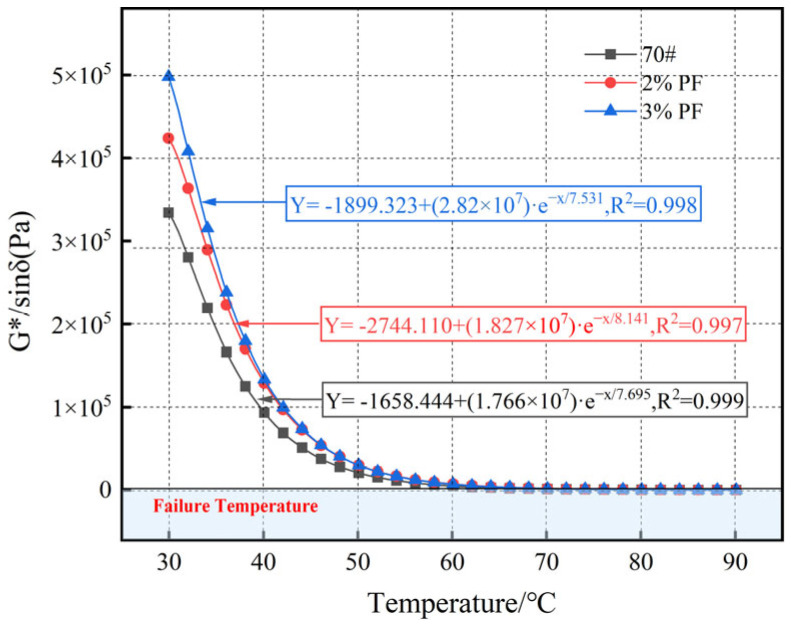
Rutting factors of different asphalts.

Figure 6 shows the trend of G*/sin*δ* with temperature changes. It can be observed that the value of G*/sin*δ* decreases with the increase in temperature and increases with the addition of PF, indicating that the addition of PF can significantly improve the rutting resistance of asphalt. The temperature corresponding to G*/sin*δ* = 1.0 kPa is the failure temperature of asphalt (details can be seen in Table 7). This suggests that phase separation has occurred in the polymer–asphalt blend system and the high-temperature rutting resistance of the modified asphalt is essentially lost. Through calculations, it was found that the failure temperature of 3% PF-modified asphalt is 5.6 °C higher than that of the base asphalt.

### 4.4. MSCR Testing

**Figure 7:**
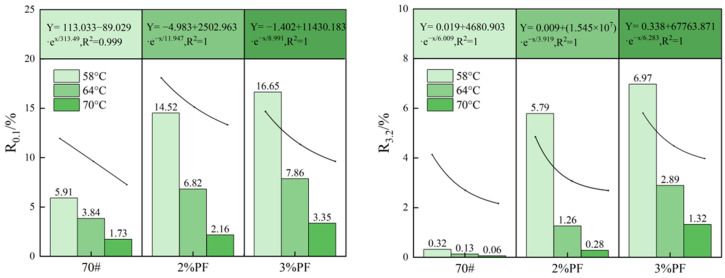
Variation of R-values with temperatures for different asphalts under two stresses.

According to the ASTM D6648 standard, the temperature corresponding to G*/sin*δ* = 1.0 kPa is defined as the failure temperature of modified asphalt. Figure 7 shows the creep recovery rate (R) of different asphalts under loads of 0.1 kPa and 3.2 kPa, respectively. When the stress load is constant, the R value of asphalt decreases with the rise in temperature. However, the R value of PF-modified asphalt achieves a significant increase compared with the base asphalt. This suggests that the addition of PF can effectively alleviate the loss of asphalt elasticity caused by the temperature rise, thereby enhancing the overall creep recovery ability. Interestingly, the nonlinear fitting of the above data reveals that the rate of reduction in the R-value of PF-modified asphalt slows down with increasing temperature when the stress is at 0.1 KPa, further confirming the contribution of PF to the asphalt’s elasticity and high-temperature stability. Meanwhile, when the applied stress increases to 3.2 KPa, the R-value of the base asphalt approaches zero, indicating near-complete failure.

**Figure 8:**
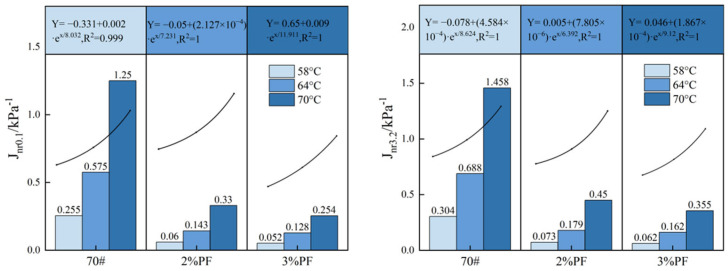
Variation of *J*_nr_ values with temperatures for different asphalts under two stresses.

As shown in Figure 8, the Jnr values of all asphalts exhibit nonlinear growth with the increase in temperature. This suggests that the fluidity of the asphalt gradually decreases and its elasticity gradually diminishes, especially at high temperatures where the viscosity of the asphalt dominates, thereby resulting in larger irreversible deformation and a higher loss of creep recovery ability [50]. The addition of PF makes the creep compliance of modified asphalt much smaller than that of base asphalt and reduces the rate of creep compliance increases when faced with temperature rises and stress increases. This is because phenol-formaldehyde resin has excellent adhesion and heat resistance, which can effectively fill the microscopic voids in the asphalt and form a strong chemical bond with them. This can enhance the shear resistance and tensile resistance of asphalt pavements, thereby reducing the deformation of pavements under repetitive loads and extending the pavements’ service lives. For them, the high-temperature deformation resistance is optimal when the PF dosage is 3%.

### 4.5. Analysis of Low-Temperature Rheological Properties

**Figure 9:**
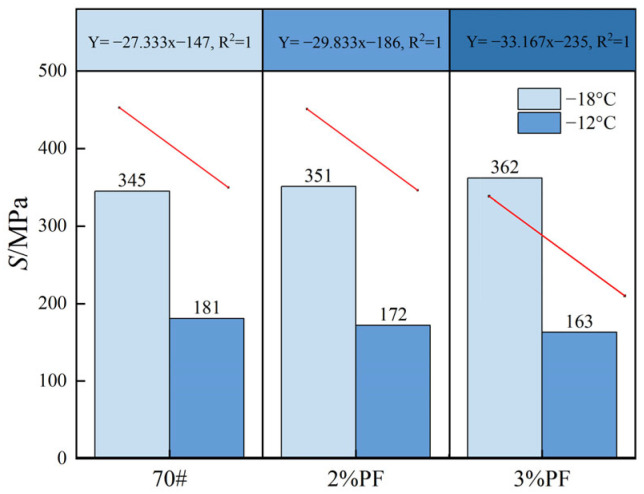
*S*-values of different asphalts.

The asphalt’s low-temperature performance and susceptibility to cracking were assessed using the BBR test, following the guidelines of the ASTM standard D6648. Figure 9 shows the *S* values of three types of asphalt at two test temperatures. It is evident that as the temperature drops from −12 °C to −18 °C the *S* value of the asphalt sharply increases, thereby indicating that the decrease in temperature causes the asphalt to become harder and more brittle. At −12 °C, the *S* values of PF-modified asphalt (142 MPa and 163 MPa) suggest that PF-modified asphalt meets the specification requirements (300 MPa). It is noteworthy that at temperatures as low as −18 °C, all three types of asphalt lose flexibility due to inherent material properties. Additionally, the brittleness of PF at low temperatures may further diminish the asphalt pavement’s flexibility and toughness.

**Figure 10:**
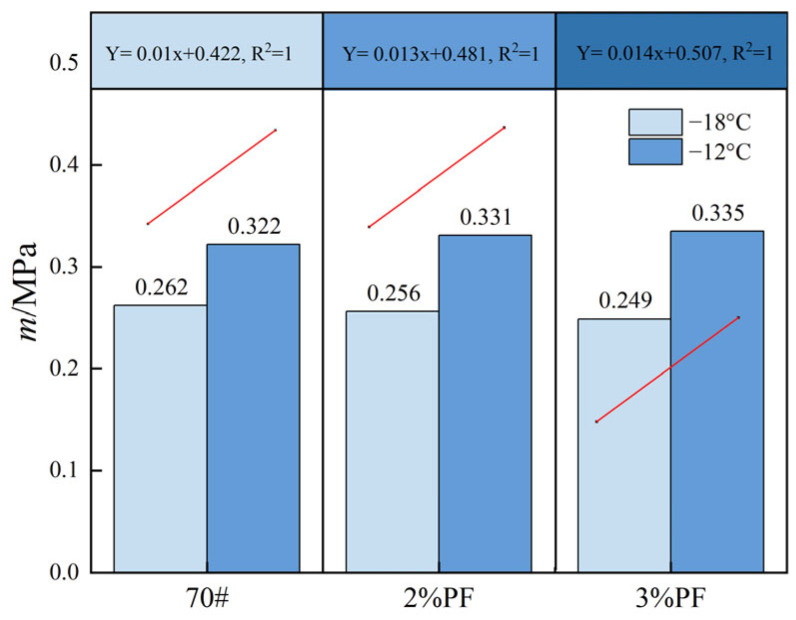
*m*-values of different asphalts.

Figure 10 presents the m values of different asphalts at two test temperatures. Consistent with the change in *S* values, the improvement effect of PF on low-temperature performance at −18 °C is not significant, with the *m* values of both 2% PF-modified asphalt and 3% PF-modified asphalt being less than that of base asphalt. This suggests that at −18 °C, the brittleness of PF impacts the creep capability of asphalt, thereby resulting in a decrease of the *m* value. However, at −12 °C, the addition of PF can increase the *m* value of asphalt to a certain extent. This is because low temperatures reduce the viscosity of asphalt, worsening the dispersion of PF in asphalt. The aggregated material that forms becomes more rigid at low temperatures, making the asphalt more brittle overall. Therefore, when the temperature drops to −18 degrees, PF molecules in the asphalt are more likely to cluster together, thereby forming larger aggregates. However, when the temperature drops to −12 degrees, the degree of PF molecule aggregation in the asphalt is lower, so the brittleness of the asphalt is relatively less.

### 4.6. PG Grading

#### 4.6.1. High-Temperature Classification

The Superpave performance grading (PG) system follows the ASTM standard D6373. To simulate the effects of short-term aging on asphalt properties, asphalt was subjected to the rolling thin film oven test (RTFOT) in accordance with the ASTM standard D2872. The PG grades are divided into six levels as follows: 46 °C, 52 °C, 58 °C, 64 °C, 70 °C, 76 °C, and 82 °C. Different asphalts are classified through these PG grades according to the standard (see Table 8).

From the PG grading of various asphalts (Table 9), it can be seen that the high-temperature PG grade for base asphalt is 70 °C, while the high-temperature PG grade for PF-modified asphalt is consistently 76 °C. This suggests that PF significantly extends the high-temperature range of asphalt use.

#### 4.6.2. Low-Temperature Classification

The low-temperature PG grades are set at −46 °C, −40 °C, −34 °C, −28 °C, −22 °C, −16 °C, and −10 °C. According to Table 10, different asphalts are classified under low-temperature PG grades, and the critical low temperatures are determined using interpolation methods. The statistical results can be found in Table 11.

### 4.7. Aging Property

**Figure 11:**
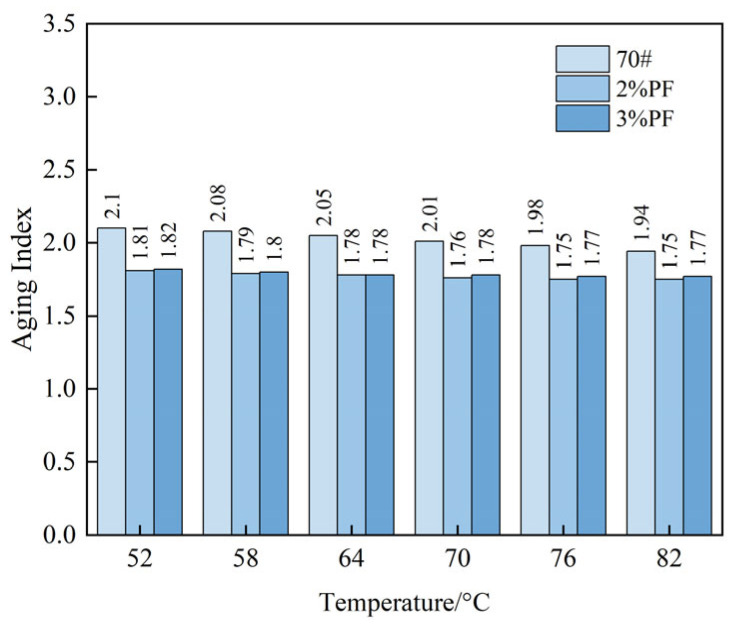
Variations of the aging index with temperatures for different asphalts.

Figure 11 shows the aging index (AI) obtained by calculating the ratio of G*/sin*δ* before and after aging. It was found that the AI value decreased with an increasing temperature within a certain range. This suggests that the temperature in the temperature scan experiment is not a key factor affecting the aging index. Furthermore, the aging index (AI) values of the base asphalt exceed those of other asphalts, peaking at 2.1 at 52 °C. This highlights the base asphalt’s heightened aging and diminished stability following short-term aging. The results show that the addition of PF can enhance the asphalt’s resistance to aging and stability. This can be attributed to the excellent thermal and chemical stability of PF, which effectively protects the asphalt from environmental factors such as oxidation that lead to degradation. Moreover, the phenolic groups within PF possess antioxidative properties that are capable of reacting with free radicals, thus reducing the quantity of free radicals in the asphalt and consequently diminishing the degree of aging of the asphalt surface.

### 4.8. Marshall Testing

**Figure 12:**
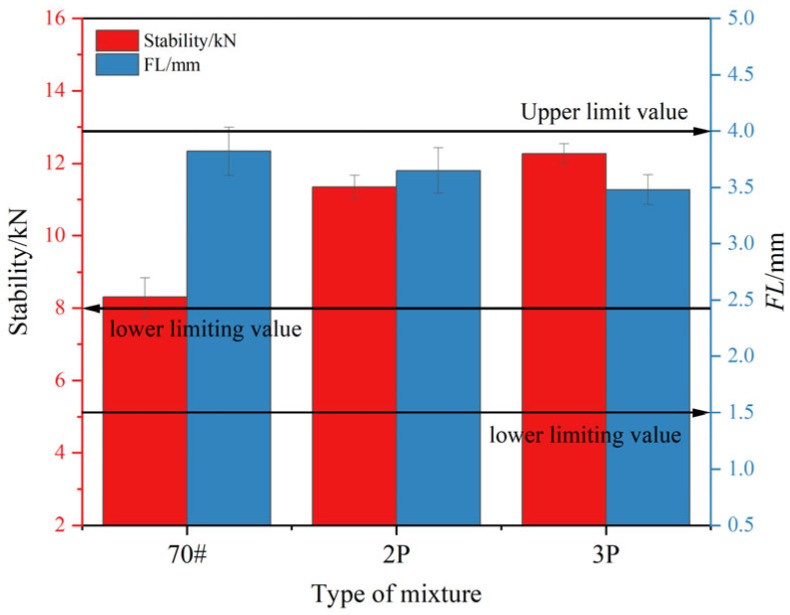
Marshall stability and flow value of different asphalt mixtures.

As shown in Figure 12, all asphalt mixtures meet the specification requirement of a Marshall stability higher than 8 kN for hot regions (ASTM D6927). Among them, the stability of the base asphalt mixture is the lowest, indicating that the addition of PF has a positive effect on the high-temperature deformation resistance and high-temperature stability of the asphalt mixture. At the same time, the stability of the 3% PF-modified asphalt mixture is higher than that of the 2% PF-modified asphalt mixture, indicating that PF has a positive response to the high-temperature performance and adhesion of the asphalt mixture within the 0–3% PF content range. By observing the deformation quantity (flow value) when different asphalt mixtures encounter damage, it was found that the content of PF is negatively correlated with the flow value. This suggests that the addition of PF can reduce the deformation of the asphalt mixture. This may be due to PF providing sufficient adhesion and a more stable structure and toughness to the asphalt.

**Figure 13:**
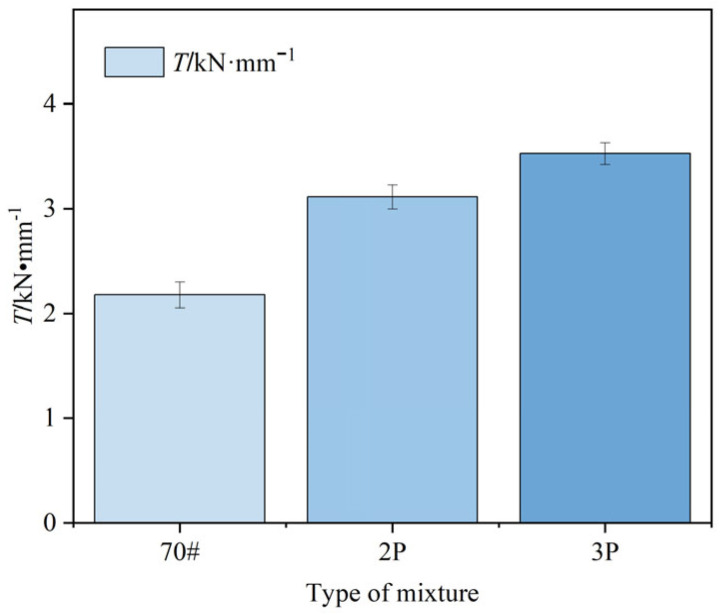
*T*-value of different asphalt mixtures.

By calculating the ratio of stability to *FL*, the Marshall modulus (*T*) can be introduced to further explore the impact of PF on the asphalt mixture. The results are shown in Figure 13. With the addition of PF and the increase in its content, the value of *T* gradually increases and the standard deviation gradually decreases. This suggests that the modified asphalt mixture with added PF has a stronger load-bearing capacity and a high-temperature resistance under the same deformation and temperature conditions.

### 4.9. Water Stability Analysis

#### 4.9.1. Immersion Marshall Testing

**Figure 14:**
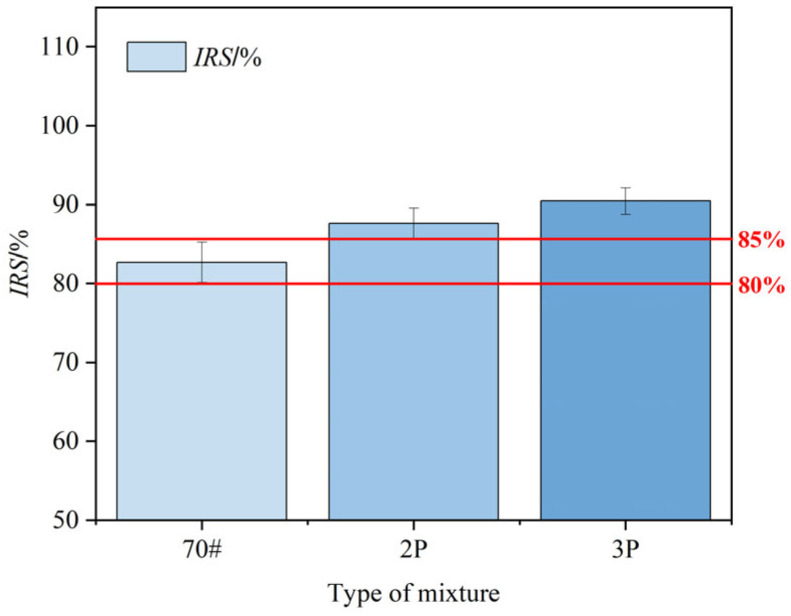
*IRS*-value of different asphalt mixtures.

The water stability of PF-modified asphalt mixtures can be evaluated by calculating the ratio of Marshall stability before and after water immersion to obtain immersion residual stability (IRS), as shown in Figure 14. It can be seen that the immersion residual stability of all kinds of asphalt mixtures meets the corresponding standards for rainy regions (the IRS of the matrix asphalt mixture is ≥80% and the IRS of the modified asphalt mixture is ≥85%). At the same time, the immersion residual stability of 2% PF- and 3% PF-modified asphalt mixtures increased by 7% and 9%, respectively, compared with the matrix asphalt. This is due to the strong water resistance of PF itself, which can effectively prevent the mixture from being eroded and damaged by water.

#### 4.9.2. Freeze–Thaw Splitting Testing

**Figure 15:**
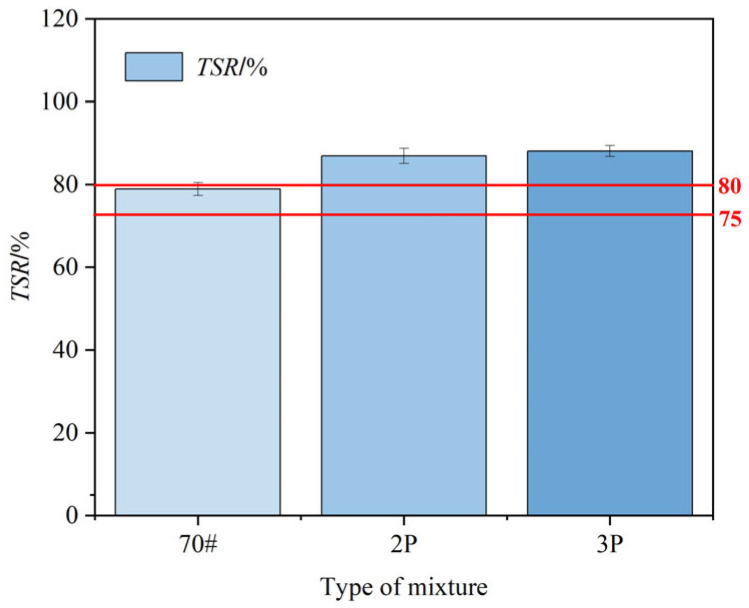
*TSR*-value of different asphalt mixtures.

By conducting indirect tensile split tests on asphalt mixtures before and after freeze–thaw treatment, the freeze–thaw split strength ratio (*TSR*) can be calculated, as shown in Figure 15. It can be seen that all types of asphalt mixtures meet the corresponding standards for rainy regions (the *TSR* of the matrix asphalt mixture is ≥75%, and the TSR of the modified asphalt mixture is ≥80%). Meanwhile, the *TSR* value increases with the increase in PF content, and the addition of 2% PF and 3% PF increases the *TSR* value of asphalt mixtures by 17.7% and 20.7%, respectively. Through comparative analysis, it is found that PF-modified asphalt mixtures have better adhesion with regard to the asphalt–aggregate system, water permeability resistance, and stripping resistance. This is attributed to PF forming a hard, heat-resistant, water-resistant, and chemically stable polymer, which helps to enhance the internal bonding strength of the mixture, thus allowing the material to better resist breaking during the freeze–thaw process.

### 4.10. FM Analysis

**Figure 16:**
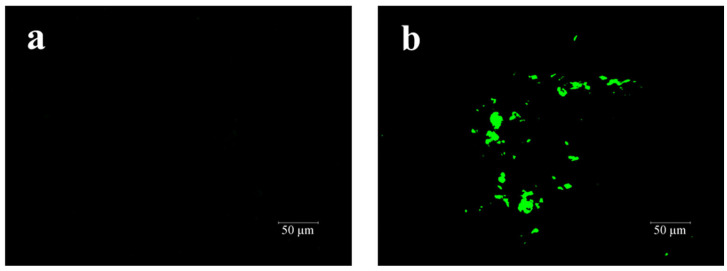
FM figures of asphalt: (**a**) base asphalt; (**b**) 3% PF-modified asphalt.

FM tests on base asphalt and 3% PF-modified asphalt were conducted to further investigate the impact and variations of PF’s three-dimensional crosslinking within the asphalt. As depicted in Figure 16, the base asphalt appears black in Figure 16a due to its lack of fluorescence. In contrast, Figure 16b displays irregularly shaped and unevenly sized PF particles uniformly dispersed throughout the asphalt, with no signs of aggregation. This dispersion of rigid three-dimensional particles can further restrict the flow of asphalt at high temperatures.

### 4.11. FT-IR Analysis

**Figure 17:**
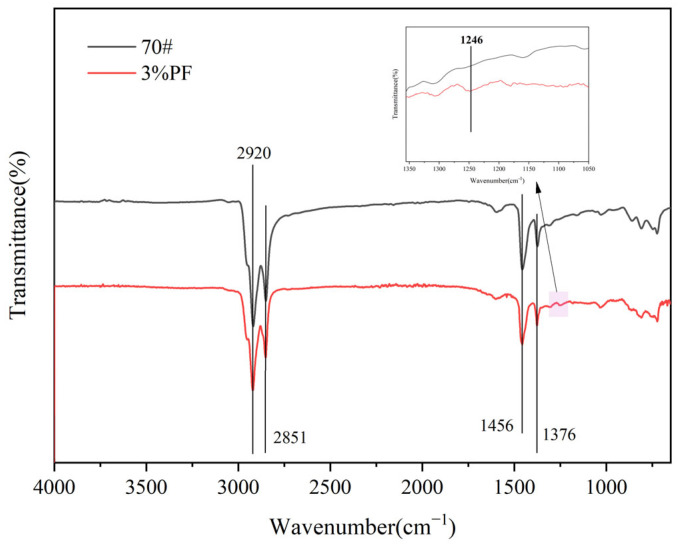
FT-IR figures of asphalts.

To better detect the impact of PF on the chemical structure and composition of asphalt, FT-IR testing was conducted on 3% PF-modified asphalt and the base 70# asphalt binder. As depicted in Figure 17, both samples exhibited absorption peaks at 2920 cm⁻^1^ and 2851 cm⁻^1^, corresponding to the asymmetric and symmetric stretching vibrations of the C-H bonds. Additionally, peaks at 1456 cm⁻^1^ and 1376 cm⁻^1^ were observed, associated with the bending vibrations of the C-H bonds and the symmetric C-H bending vibrations of methyl (CH_3_) groups, respectively. Of particular note is the peak at 1246 cm⁻^1^ (highlighted in the inset of the figure) observed for the 3% PF-modified asphalt, which is attributed to the characteristic structures of PF such as the stretching vibrations of the C-O-C bonds in aromatic rings. Overall, the FT-IR test results suggest that no significant chemical reactions occur between PF and the 70# base asphalt binder; PF predominantly exists in a physically dissolved state within the asphalt.

## 5. Conclusions

This study selected PF as a modifier for 70# base asphalt. Through comprehensive analysis including three major indicator tests and viscosity testing, the optimal PF content range was determined. The research investigated how varying PF contents influenced the high-temperature and low-temperature rheological properties as well as the anti-aging performance of the base asphalt. Furthermore, it delved into the microstructural morphology and interaction mechanisms of PF within the asphalt binder. An innovative aspect of this study involved a concurrent analysis of the impact mechanism of PF-modified asphalt binders on the stability and resistance to water damage of asphalt mixtures. Based on the results, the following conclusions were drawn:

(1) Adjusting PF content modulates the conventional physical properties of asphalt. Increased PF content decreases penetration and ductility while raising the softening point and viscosity. PF-modified asphalts with 2% and 3% PF showcased the most notable modification effects.
(2) PF contributes to delaying the transition of asphalt from an elastic state to a viscous state, with 3% PF raising the destruction temperature of the base asphalt by 5.6 °C. Owing to PF’s exceptional mechanical properties, it greatly enhances the shear and tensile resistance of the base asphalt.
(3) The elevation of the high-temperature performance grade (PG) from 70 °C for the base asphalt to 76 °C for PF-modified asphalt highlights the potential of PF to extend the operational range of asphalt under high-temperature conditions.
(4) PF does not significantly enhance the low-temperature crack resistance of asphalt binders.
(5) The high viscosity and adhesive properties of PF-modified asphalt reduce porosity within the mixture, enhancing adhesion between different components and improving resistance to high-temperature deformation as well as stripping in the mixture.
(6) The uniform dispersion of PF particles without aggregation contributes to limiting the asphalt flow at high temperatures and inhibiting oxygen penetration.

In summary, this study reveals the potential mechanisms underlying PF-controlled rheological behaviors and the macroscopic mechanical properties of asphalt mixtures. It provides valuable insights into the construction of asphalt pavements tailored to withstand increased traffic loads and harsh environmental conditions. Future research will focus on understanding the interfacial mechanisms between PF and asphalt as well as elucidating the viscoelastic response to PF degradation and asphalt oxidation interactions.

## Figures and Tables

**Table 1:** Parameter index of 70# matrix asphalt used in the experiment.

Index	Test Result	Specification Requirements	Test Method
Penetration (100 g 5 s 25 °C) (0.1 mm)	67.2	60–80	ASTM D5 [35]
Ductility (5 cm/min 15 °C) (cm)	150	>100	ASTM D113 [36]
Softening point (°C)	47.3	>46	ASTM D36 [37]
Kinematic viscosity (60 °C) (Pa·S)	110	----	ASTM D4402 [38]

**Table 2:** PF parameter indicators.

Technical Index	Unit	Test Result
Specific surface area	m^2^/g	140
Fineness	mesh	>200
Moisture content	%	<4.0
Flow length	mm	25~40
Melting point	°C	85~92
Free phenol content	%	3.0~4.0

**Table 3:** Coarse aggregate performance index.

Technical Index	Unit	Test Result	Specification Requirements	Test Method
Los Angeles abrasion loss	%	16.7	≤30	ASTM C131 [39]
Crushing value	%	20.5	≤28	ASTM D5821 [40]
Soundness	%	3.72	≤12	ASTM C88 [41]
Soft stone content	%	1.33	≤3.0	-
Needle and flake content	%	10.13	≤15	ASTM D4792 [42]
Particle size > 9.5 mm	%	9.54	≤12	ASTM C136 [43]
Particle size < 9.5 mm	%	12.66	≤18	ASTM C136 [43]

**Table 4:** Fine aggregate performance index.

Technical Index	Unit	Test Result	Specification Requirements	Test Method
Mud content (<0.075 mm)	%	2.37	≤3	ASTM D1140 [44]
Apparent specific gravity	-	2.64	≥2.50	ASTM C128 [45]
Sand equivalent	%	77.81	≥30	ASTM D2419 [46]
Soundness	%	4.82	≤12	ASTM C88

**Table 5:** Density index of different grades of aggregates.

Technical Index	Unit	10–20 Gears	5–10 Gears	Aggregate Chips
Apparent density	t/m^3^	2.71	2.786	2.635
Water content	%	0.32	0.69	0.48
Gross volume relative density	t/m^3^	2.69	2.734	2.602

**Table 6:** Marshall test index at optimal asphalt dosage.

Index	70#	2% PF	3% PF	Specification
*OAC*/%	4.77	5.03	4.97	----
*γ_f_*	2.14	2.38	2.38	----
*VV*/%	5.52	5.22	4.78	4~6
*MS*/kN	8.28	11.35	12.26	≥8
*FL*/mm	3.78	3.65	3.48	1.5~4
*VMA*/%	17.32	15.79	15.32	≥14
*VFA*/%	66.32	67.35	68.28	65~75

**Table 7:** Critical temperature of different asphalts.

Asphalt Type	Critical Temperature/°C
70#	69.5
2% PF	74.2
3% PF	75.1

**Table 8:** Superpave’s asphalt high temperature PG grading standards.

Asphalt State	Test Method	PG Grading Basis
Not aged	DSR	G*/sin*δ* ≥ 1.0 kPa
Short term aging	DSR	G*/sin*δ* ≥ 2.2 kPa

**Table 9:** High temperature PG classification of different asphalts.

Asphalt Type	Temperature/°C	G*/sin*δ*/kPa	Asphalt State	PG	High Temperature PG Level/°C
70#	64	2.92	Before aging	70	70
	70	1.31			
	70	2.89	After aging	76	
	76	1.20			
2% PF	70	2.10	Before aging	76	76
	76	1.15			
	70	1.81	After aging	76	
	76	3.85			
3% PF	70	2.08	Before aging	76	76
	76	1.09			
	76	1.59	After aging	76	
	82	0.75			

**Table 10:** Superpave’s asphalt low temperature PG grading standards.

Asphalt State	Test Method	PG Grading Basis
After long-term aging	BBR	*S* ≤ 300 MPa, *m* ≥ 0.3

**Table 11:** Low temperature PG classification of different asphalts.

Asphalt Type	*S*-Value Critical Temperature/°C	*m*-Value Critical Temperature/°C	Low Temperature PG Level/°C	Low Temperature PG Continuous Grading/°C
70#	−25.3	−22.5	−22	−22.5
2% PF	−25.3	−22.6	−22	−22.6
3% PF	−24.6	−22.5	−22	−22.5

## Data Availability

The data presented in this study are available on request from the corresponding author.
